# Serum Disease-Specific IgG Fc Glycosylation as Potential Biomarkers for Nonproliferative and Proliferative Diabetic Retinopathy Using Mass Spectrometry

**DOI:** 10.1016/j.mcpro.2025.100967

**Published:** 2025-04-09

**Authors:** Yishuang Mao, Jiyun Zhang, Yixin Zhang, Bojie Hu, Yuhua Hao, Zhonghao Yuan, Xufeng Zhao, Yusong Wang, Zhangwanyu Wei, Weihong Yu, Zhili Li

**Affiliations:** 1 Department of Ophthalmology, Peking Union Medical College Hospital, Beijing, China; 2 Department of Biophysics and Structural Biology, Institute of Basic Medical Sciences, Chinese Academy of Medical Sciences & School of Basic Medicine, Peking Union Medical College, Beijing, China; 3 Beijing Tongren Eye Center, Beijing Tongren Hospital of Capital Medical University, Beijing, China; 4 Department of Retina, Tianjin Medical University Eye Hospital, Tianjin, China; 5 Department of Ophthalmology, The Fourth Hospital of Hebei Medical University, Shijiazhuang, China; 6 Key Laboratory of Ocular Fundus Diseases, Chinese Academy of Medical Sciences, Beijing, China

**Keywords:** biomarkers, diabetic retinopathy, IgG N-glycosylation, mass spectrometry

## Abstract

This study investigated the potential of serum disease-specific immunoglobulin G (DSIgG) crystallizable fragment (Fc) N-glycosylation as a diagnostic biomarker for the identification of nonproliferative and proliferative diabetic retinopathy (DR). A total of 160 patients were enrolled and categorized into three groups according to clinical diagnosis: non-diabetic retinopathy (NDR, n = 47); nonproliferative diabetic retinopathy (NPDR, n = 51); and proliferative diabetic retinopathy (PDR, n = 62). Gel electrophoresis was performed to separate IgG from morning fasting blood samples and polyaniline magnetic nanomaterials (Fe_3_O_4_@PANI) were used to enrich IgG N-glycopeptides from tryptic digestion. Matrix-assisted laser desorption ionization time-of-flight mass spectrometry (MALDI ToF MS) was used to detect the IgG N-glycopeptides. Nine DSIgG N-glycopeptide ratios were significantly different among NDR, NPDR, and PDR groups. There are six glycopeptide ratios available to classify mild, moderate, and severe NPDR. Moreover, four glycopeptide ratios could identify patients with or without diabetic macular edema (DME). The prediction model exhibited good discriminatory performance in distinguishing patients with DR or NDR (AUC = 0.8347), NPDR or PDR (AUC = 0.7002), mild/moderate or severe NPDR (AUC = 0.8059), and with or without DME (AUC = 0.7846). DSIgG Fc N-glycosylation ratios were closely associated with different stages of DR and may be used as potential biomarkers for the early diagnosis of NDR, NPDR, and PDR.

## Highlights

• A multicentric research with an ideal sample size, and the inclusion of patients with refined demographic characteristics and clinical staging of diabetic retinopathy.
• A method for the rapid analysis of the N-glycosylation of serum IgG in patients with diabetic retinopathy (DR).
• The prediction model exhibited good discriminatory performance in distinguishing patients with different stages of DR and may be used as a potential biomarker in future clinical practice.

## Graphical Abstract

**Figure:**
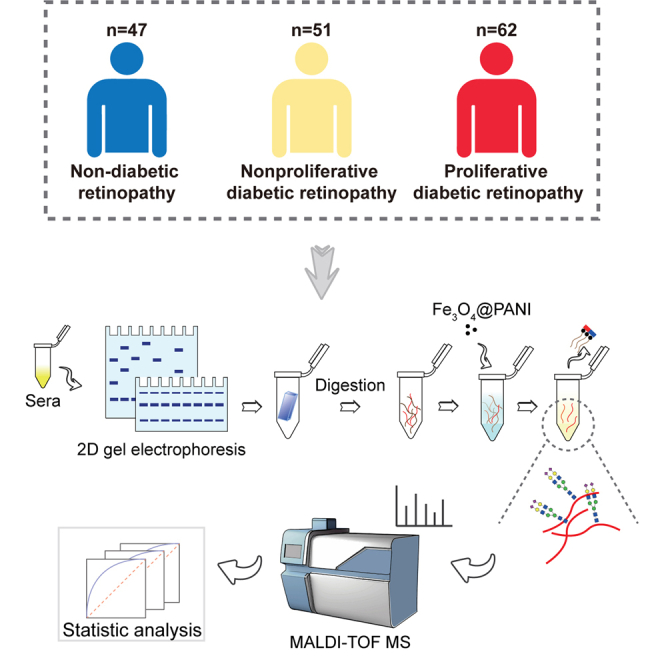

## In Brief

In this study, a total of 160 patients with NDR, NPDR, or PDR were enrolled. DSIgG Fc N-glycosylation was detected using MALDI ToF MS. We identified key N-glycopeptide ratios that significantly differ among DR stages and DME, suggesting their potential as diagnostic biomarkers for DR detection.

Diabetes mellitus (DM) refers to several disorders of carbohydrate metabolism characterized by hyperglycemia, which are associated with either a relative or an absolute impairment of insulin secretion, with varying degrees of insulin resistance. Diabetic retinopathy (DR) is a microvascular complication that occurs in about 30%∼40% of diabetic individuals (1). DR is one of the most important causes of vision loss worldwide and the leading cause of vision impairment in patients aged 25 to 74 years (2). Hyperglycemia activates inflammatory processes leading to oxidative stress, vascular leakage, and eventually ganglion cell loss in the retina (3). DR is classified into nondiabetic retinopathy (NDR); mild, moderate, and severe nonproliferative diabetic retinopathy (NPDR); and proliferative diabetic retinopathy (PDR) (4). Individualized treatment plans, including intravitreal injection of anti-vascular endothelial growth factor (anti-VEGF) and laser photocoagulation, are applied based on the course of the disease.

DR may be asymptomatic for years, even in advanced stages, causing a dreadful outcome for patients. Because of this, using new methods for early screening is essential for the prognoses of DR patients (5). Nowadays, DR screening depends heavily on tests such as color fundus photography, optical coherence tomography (OCT), and slit lamp examinations by experienced ophthalmologists, which is both time and financially consuming. Therefore, an ideal diagnostic tool that is non-invasive, highly accurate sensitive, and easy to implement is in urgent demand. Blood biomarkers, for instance, are excellent candidates.

Our previous studies showed that serum immune-inflammation-related protein complexes (IIRPCs) are strongly associated with DR disease status (6, 7). It has been proved that glycosylation of disease-specific immunoglobulin G (DSIgG), one of the major IIRPC components, is linked to abundant diseases, such as diabetes (8), neurologic diseases (9), and auto-immune diseases (10), for IgG glycosylation alters the proinflammatory and anti-inflammatory balance in the human body.

IgG is the most abundant glycoprotein in blood. They have exceptional temporal stability, thus underling superb diagnostic potential. IgG has two functional domains: an antigen-binding fragment (Fab) and a crystallizable fragment (Fc). The Fc domain contains a highly conserved glycosylation site at asparagine 2974. Various glycan structures can be attached to the spot, and affinity to Fc receptors may be altered in the meantime (11). In previous studies, bisecting N-acetylglucosamine (GlcNAc) and fucosylation exert a pro-inflammatory effect, while the addition of galactose or sialic acid has an anti-inflammatory effect (3).

Our previous study proved that DSIgG Fc N-glycosylation ratios were associated with NPDR and can be used as potential biomarkers for the early diagnosis of NPDR (12). This gave us confidence to further our research in the field of IgG glycosylation in DR patients of all stages and different clinical characteristics, by measuring the amount and ratio of specific IgG glycans. This can give clinicians a new perspective in understanding the pathology underlying the onset and development of DR and enable the discovery of new blood biomarkers that could be used in DR clinical screening and monitoring.

## Method

### Patient Enrollment and Study Design

From 2021 to 2022, patients with DM were recruited from three respective ophthalmology departments. Patients with DM were examined by senior ophthalmologists and were diagnosed with NDR, NPDR, or PDR, with or without DME after a thorough inspection. The criteria for patient inclusion were as follows: (a) patients who met the diagnostic criteria of type 2 DM issued by WHO in 1999 and (b) patients with NDR, NPDR, and PDR who met the 2002 International Classification of Diabetic Retinopathy diagnostic criteria. NDR refers to patients who have DM but show no sign of diabetic retinopathy. NPDR includes mild, moderate, and severe NPDR. Mild NPDR manifests as microaneurysms only. Moderate NPDR is characterized by more than just microaneurysms but is less critical than severe NPDR. Patients were diagnosed with severe NPDR if they exhibited any of the following: more than 20 intraretinal hemorrhages in each of 4 quadrants; definite venous beading in more than two quadrants; prominent intraretinal microvascular abnormalities in more than one quadrant and no signs of proliferative retinopathy. Patients with PDR exhibit neovascularization of the retina or iris, or preretinal or vitreous hemorrhages (5).

**Fig. 1:**
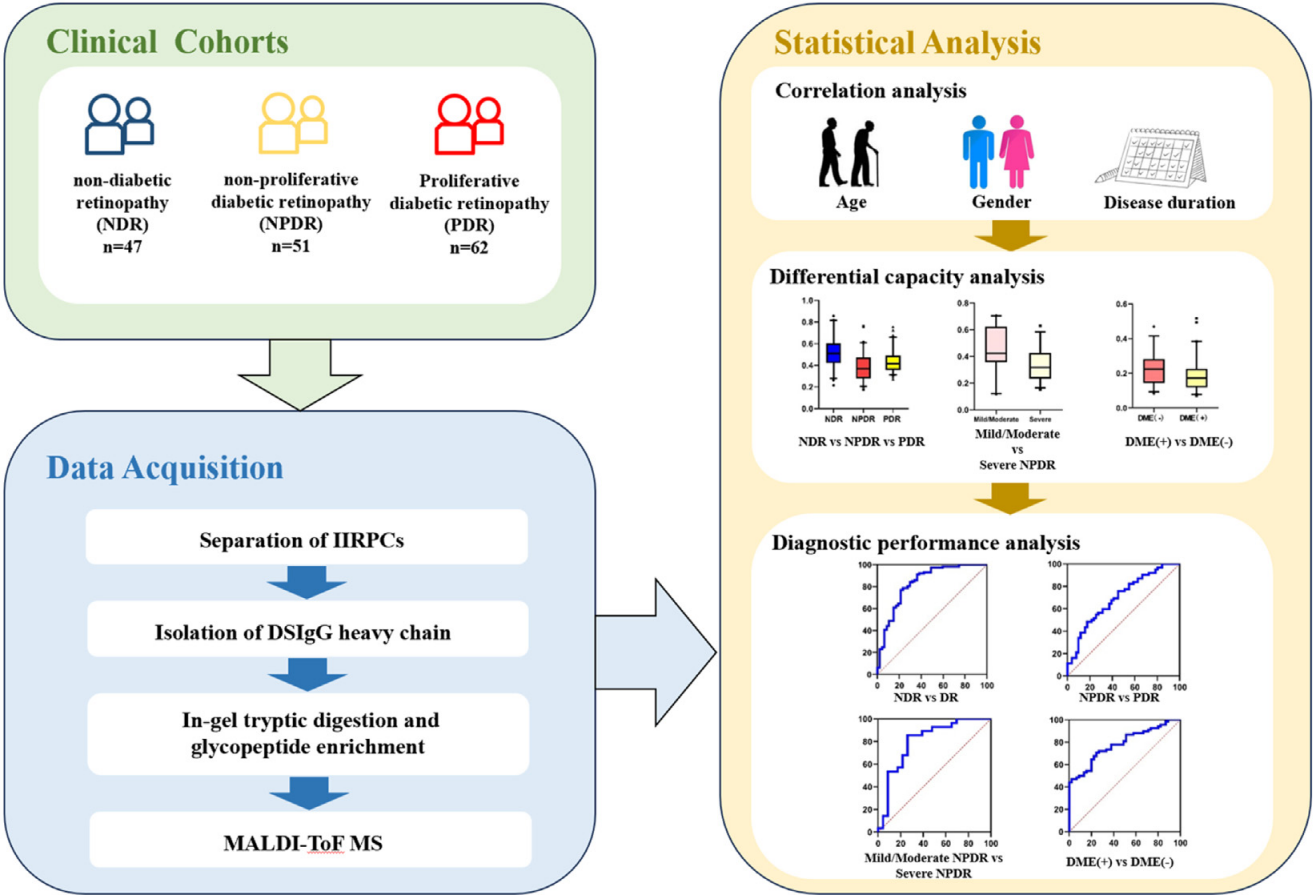
**Workflow of this study.** DME, diabetic macular edema; IIRPs, immune-inflammation-related protein complexes; NDR, non-diabetic retinopathy; NPDR, non-proliferative diabetic retinopathy; PDR, proliferative diabetic retinopathy.

The criteria for patient exclusion were as follows: (a) patients who had type 1 DM; (b) patients who had severe systemic diseases, including hepatic and renal insufficiency, cardiovascular and pulmonary insufficiency, autoimmune diseases, psychiatric abnormalities, and malignant tumors, and (c) patients who had other ocular diseases that may interfere with the results, such as uveitis and infectious ocular diseases. Consent forms were signed prior to enrollment. Patients’ demographic characteristics, including age, gender, duration of DM, fasting blood glucose, serum creatinine levels, and history of hypertensive and renal diseases, were collected. The study design and workflow is displayed in Figure 1. The study was conducted according to the Declaration of Helsinki and approved by the Ethics Review Committee of Peking Union Medical College Hospital (No. JS-3253).

### Sample Collection

Morning fasting peripheral venous blood was collected from all participants and stored at −80 °C freezer immediately.

### Separation of IIRPCs

IIRPCs were separated using gel electrophoresis (13). Every nine serum samples were experimented simultaneously with one quality control (QC) sample (serum of 6 random pooled mixed samples). IIRPCs were separated by a 4%–10% native-PAGE (polyacrylamide gel electrophoresis) gradient with 10 μl serum loaded (14). Each gel was electrophoresed at a constant current of 10 mA for 1.5 h, then converted to 25 mA for 3 h, followed by staining with Coomassie brilliant blue and decolorization with ultrapure water.

### Isolation of DSIgG Heavy Chain

The excised IIRPCs gel bands were reduced in-gel with 200 μl of 0.2 M dithiothreitol (Calbiochem, Merck) at 37 °C for 45 min, followed by alkylation with 200 μl of 0.32 M iodoacetamide (Vetec reagent grade, 99%, Sigma-Aldrich) in the dark for 30 min (15). DSIgG heavy chains were obtained after rinsing with ultrapure water and isolation by SDS-PAGE (sodium dodecyl sulfate-polyacrylamide gel electrophoresis). Electrophoresis was conducted at 60 V for 45 min, then 120 V for 1.5 h, followed by overnight staining with Coomassie brilliant blue and decolorization with ultrapure water.

### In-Gel Tryptic Digestion and Glycopeptide Enrichment

Bands of the DSIgG heavy chains in the SDS-PAGE gel were excised and destained repeatedly until colorless, then acetonitrile (ACN) was used for dehydration. The supernatants were aspirated and vacuumed dry. The incubation process was conducted with 10 μl of 12.5 ng μL^−1^ trypsin (sequencing grade modified, Roche Diagnostics) solution, prepared with 25 mM ammonium bicarbonate at 4 °C for 1 h 150 μl of 25 mM ammonium bicarbonate was added and together incubated for 16 h at 37 °C, followed by aspiration and vacuuming. After that, magnetic polyaniline nanomaterial Fe_3_O_4_ @PANI was dispersed in 80% ACN by ultrasonication to prepare an enrichment solution with a concentration of 2 mg mL^−1^. A total of 100 μl of the enrichment solution was placed into tryptic digests, followed by shaking for 1 h. After magnetic separation, the supernatant was discarded. Rinsed 3 times with 80% ACN, 100 μl of 0.025% ammonia eluate was added to each tube and magnetically separated after shaking for 45 min. The supernatant was collected and lyophilized for mass spectrometry analysis (16).

### Mass Spectrometry Detection

All experiments were performed using fleXtreme matrix-assisted laser desorption ionization time-of-flight mass spectrometry (MALDI ToF MS, Bruker Daltonics). A total of 0.35 μl of solution was precisely placed on the MALDI target plate, air-dried at room temperature, and 0.35 μl of 20 μg μL^−1^ 2,5-dihydroxybenzoic acid (50% ACN/0.1% TFA) was added. In positive ion mode, the m/z range was 500 to 5000. GlycoMod (http://web.expasy.org/glycomod/) was used to predict possible glycostructures based on experimentally determined glycopeptide masses (Supplemental Table S1). Monoisotopic masses of glycopeptides with signal/noise (S/N) of >3 were extracted for further analysis.

### Statistical Analysis

FlexAnalysis and Matlab were used to extract the signal intensity of each glycopeptide peak with a signal-to-noise threshold of >3.0. The extracted data were further analyzed, and glycopeptide ratios were calculated. After integrating patients’ clinical information, the data were further categorized according to the 2002 International Classification of Diabetic Retinopathy diagnostic criteria into three groups (NDR, NPDR, and PDR). GraphPad Prism 8.0, SPSS 23.0, and Origin 2021 were used to analyze the following data. For continuous variables adhering to a normal distribution, one-way ANOVA was applied. The Kruskal-Wallis test and the two-sided Mann-Whitney U test were applied to continuous variables that did not follow a normal distribution. The chi-square test was used in the analysis of categorical variables. Correlations between DSIgG glycopeptide ratios were investigated using Spearman correlation analysis. Binary logistic regression was used to calculate the joint probability of DSIgG’s diagnostic efficacy, while ROC curves assessed the diagnostic ability of the glycopeptide ratios. Two-sided *p*-values less than 0.05 were considered statistically significant in this study.

## Results

### Clinical Characteristics

A total of 160 patient samples were categorized into three groups based on clinical diagnosis: NDR (n = 47), NPDR (n = 51) and PDR (n = 62). There were no significant differences in gender, age, prevalence of hypertension and renal disease among the three groups (*p* > 0.05). However, there were significant differences in the duration of DM (*p* = 0.008), fasting blood glucose (FBG, *p* = 0.001), serum creatinine level (Scr, *p* = 0.005) and diabetic macular edema (DME, *p* = 0.000). Detailed baseline demographic and clinical characteristics are listed in Table 1.

**Table 1:** Baseline demographic and clinical characteristics of patients in this study.

	NDR (n = 47)	NPDR (n = 51)	PDR (n = 62)	*p* value
Age (years)	64 (58,70)	65 (58,71)	61 (53,67)	0.114
Gender (male/female)	26/21	19/32	32/30	0.158
Duration of DM (years)	11.3 (7.0,15.0)	15.0 (10.0,20.0)	15.0 (11.0,20.0)	0.008
FBG (mmol/L)	8.11 (±1.68)	8.95 (±2.00)	9.79 (±3.01)	0.001^a^
Scr (mmol/L)	74.16 (±17.5)	103.55 (±62.42)	116.55 (±140.74)	0.005^a^
HTN, n (%)	25 (53.19)	26 (50.98)	28 (45.16)	0.682
Renal disease, n (%)	1 (2.23)	5 (9.80)	7 (11.29)	0.193
DME, n (%)	0 (0.00)	17 (33.33)	28 (45.16)	0.000^a^

DM, diabetes mellitus; DME, diabetic macular edema; FBG, fasting blood glucose; HTN, hypertension; Scr, serum creatinine level.

a *p* < 0.05.

### DSIgG Fc Glycopeptides Profiling

**Fig. 2:**
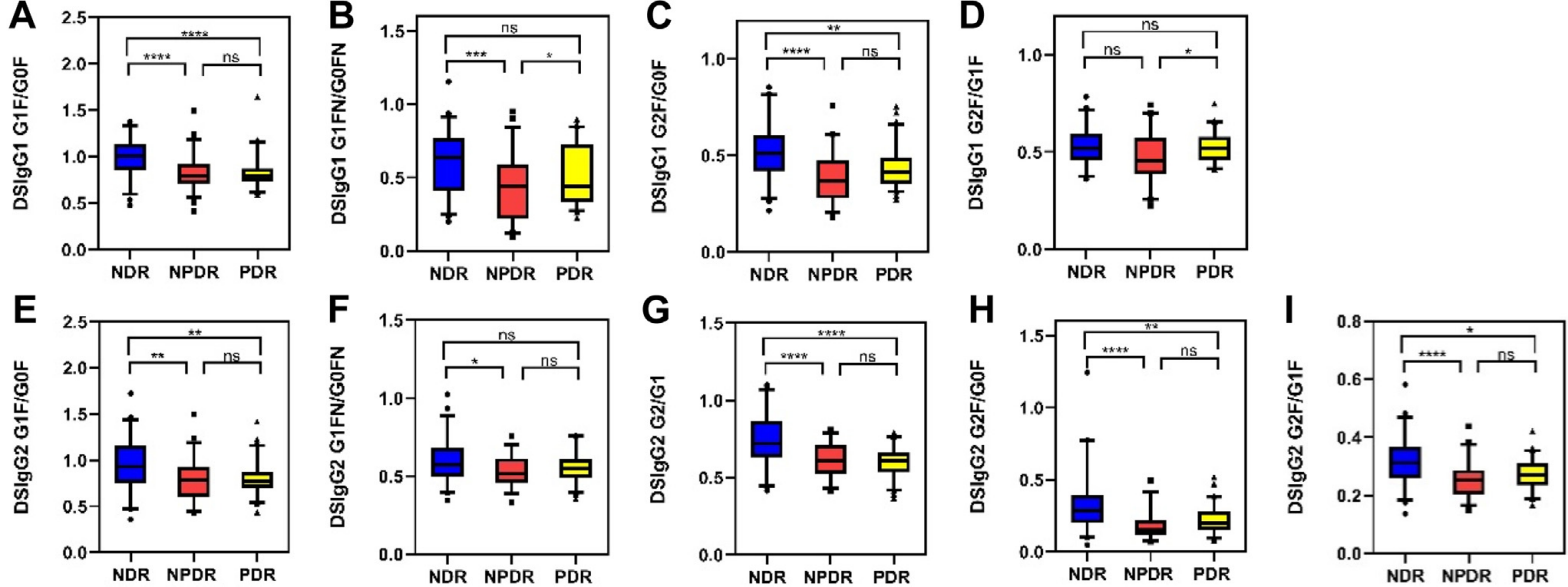
**Comparison of the DSIgG Fc glycopeptide ratios between NDR, NPDR, and PDR groups.***A*, DSIgG1 G1F/G0F; *B*, DSIgG1 G1FN/G0FN; *C*, DSIgG1 G2F/G0F; *D*, DSIgG1 G2F/G1F; *E*, DSIgG2 G1F/G0F; *F*, DSIgG2 G1FN/G0FN; *G*, DSIgG2 G2/G1; *H*, DSIgG2 G2F/G0F; *I*, DSIgG2 G2F/G1F.

DSIgG glycopeptides were detected utilizing MALDI ToF MS in 160 patients’ serum samples and 25 QC samples. A total of 14 glycopeptides were detected, including 6 DSIgG1 glycopeptides and 8 DSIgG2 glycopeptides. Structures and m/z values of detected DSIgG N-glycopeptides are shown in Supplemental Table S2. The relative quantification of detected DSIgG N-glycopeptides in each sample is shown in Supplemental Table S3. The intensity of the different glycopeptides’ peaks varied within the three groups. Representative positive ion mass spectra of DSIgG Fc N-glycopeptides from three individuals from the respective groups, NDR (Fig. 2*A*), NPDR (Fig. 2*B*), and PDR (Fig. 2*C*), are shown in Supplemental Fig. S1.

Previous studies using serum metabolomics have shown that highly correlated glycopeptide pairs, especially the ones in which glycan structures are only one enzymatic step apart in the known IgG glycosylation pathway, represent glycosylation enzymatic reactions (17). Thus, 17 glycopeptide ratios (DSIgG1: G0FN/G0F, G1F/G0F, G1FN/G0FN, G1FN/G1F, G2F/G0F, G2F/G1F; DSIgG2: G0FN/G0F, G1F/G0F, G1F/G1, G1N/G1, G1FN/G0FN, G1FN/G1F, G1FN/G1N, G2/G1, G2F/G0F, G2F/G1F, and G2F/G2) were selected, each meeting the criteria of RSDs < 20% in QC samples, indicating that the measurement in these glycopeptide ratios was stable and reliable for further analysis.

### Variances of Glycopeptide Ratios

#### Variances of Glycopeptide Ratios with Gender, Age, and DM Disease Duration

There were no significant differences between male and female patients in each group, respectively.

DSIgG2 G1FN/G0FN was found to have a negative correlation with age in NDR (*p* = 0.004, r = −0.415), NPDR (*p* = 0.032, r = −0.300) and PDR (*p* = 0.004, r = −0.300) groups using Pearson correlation analysis. DSIgG2 G2F/G2 was found to be positively related to age in the NPDR group (*p* = 0.013, r = +0.345). DSIgG2 G2/G1 and DSIgG2 G2F/G1F were found to be negatively related to age in the PDR group (*p* = 0.033, r = −0.217; *p* = 0.045, r = −0.256).

There were no significant correlations between DM duration and glycopeptide ratios in the NDR group and the PDR group. In the NPDR group, DSIGg2 G1F/G0F (*p* = 0.012, r = −0.349), DSIGg2 G1FN/G0FN (*p* = 0.018, r = −0.331), DSIGg2 G2F/G0F (*p* = 0.021, r = −0.323) and DSIGg2 G2F/G2 (*p* = 0.032, r = −0.301) were found to have negative correlation with DM disease duration.

A lower tendency of galactosylation and a higher tendency of fucosylation indicate that aging and a prolonged disease course exhibit a higher state of inflammatory response in this scenario.

#### Variances of Glycopeptide Ratios Between Patients With NDR, NPDR, and PDR

The correlation of glycopeptide ratios varied among patients with NDR, NPDR, and PDR (Supplemental Fig. S2). Significant differences in 9 DSIgG N-glycopeptide ratios (DSIgG1: G1F/G0F, G1FN/G0FN, G2F/G0F, G2F/G1F; DSIgG2: G1F/G0F, G1FN/G0FN, G2/G1, G2F/G0F, G2F/G1F) were found between NDR, NPDR and PDR groups and are displayed in Figure 2.

Eight glycopeptide ratios (DSIgG1: G1F/G0F, G1FN/G0FN, G2F/G0F; DSIgG2: G1F/G0F, G1FN/G0FN, G2/G1, G2F/G0F, G2F/G1F) were found to be significantly different between NDR and NPDR groups. Six glycopeptide ratios (DSIgG1: G1F/G0F, G2F/G0F; DSIgG2: G1F/G0F, G2/G1, G2F/G0F, G2F/G1F) were found to be significantly different between NDR and PDR groups. Two glycopeptide ratios (DSIgG1: G1FN/G0FN, G2F/G1F) were found to be significantly different between NPDR and PDR groups.

Galactosylation is thought to have anti-inflammatory effects on IgG (18). It was found that patients with NDR have the highest tendency of galactosylation, while patients with NPDR have the lowest. This suggests that patients with NPDR exhibit the highest level of inflammatory response, while NDR patients present the lowest.

#### Variances of Glycopeptide Ratios Between Patients With Mild, Moderate, and Severe NPDR

Severe NPDR has a high risk of progressing into PDR. More active interventions, such as panretenial photocoagulation (PRP) in patients with severe NPDR, may prevent disease progression and avoid severe patient outcomes, including neovascularization, vitreous hemorrhage, and retinal detachment. Thus, it is of high importance to achieve early detection and identification of severe NPDR through accessible measures, enabling timely intervention and a better prognosis for patients (19).

**Fig. 3:**
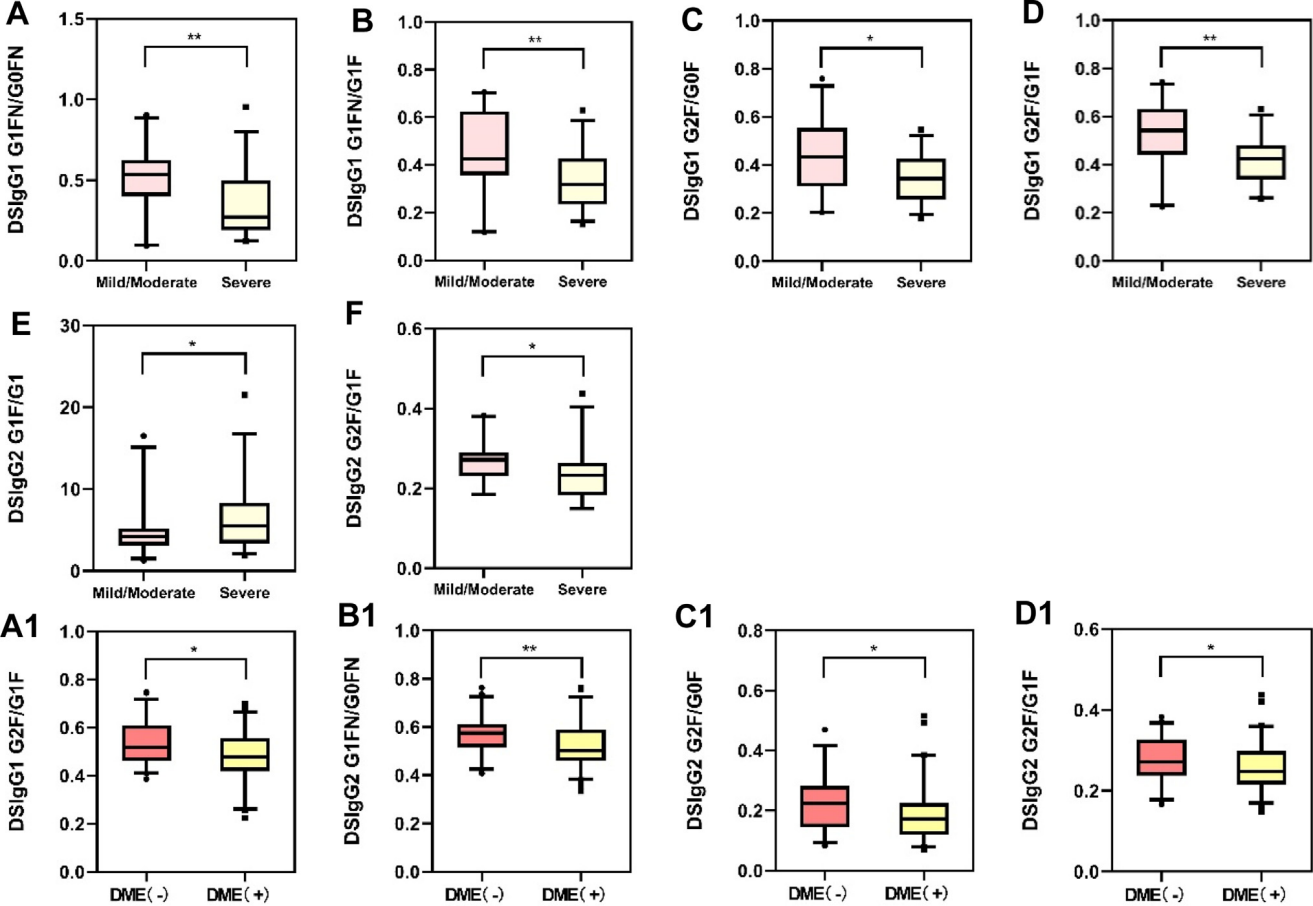
**Comparison of the DSIgG Fc glycopeptide ratios.***A*–*F*, comparison of the DSIgG Fc glycopeptide ratios between mild/moderate and severe NPDR. *A*, IgG1 G1FN/G0FN; *B*, IgG1 G1FN/G1F; *C*, IgG1 G2F/G0F; *D*, IgG1 G2F/G1F; *E*, IgG2 G1F/G1; *F*, IgG2 G2F/G1F. *A1*–*D1*, comparison of the DSIgG Fc glycopeptide ratios between patients with or with out DME. *A1*, DSIgG1 G2F/G1F; *B1*, DSIgG2 G1FN/G0FN; *C1*, DSIgG2 G2F/G0F; *D1*, DSIgG2 G2F/G1F. ns, no significance; ∗*p* < 0.05; ∗∗*p* < 0.01; ∗∗∗*p* < 0.001; ∗∗∗∗*p* < 0.0001. The p-values were calculated by the two-sided Mann-Whitney U test.

Within the NPDR group, 29 patients were diagnosed with severe NPDR among the 51 patients in total. Six glycopeptide ratios (DSIgG1 G1FN/G0FN, G1FN/G1F, G2F/G0F, G2F/G1F; DSIgG2 G1F/G1, G2F/G1F) were found to be significantly different between mild, moderate NPDR patients compared with severe NPDR patients and shown in Figure 3. Patients with severe NPDR have a lower tendency of galactosylation and a higher tendency for fucosylation, demonstrating that patients with severe NPDR manifest a higher status of inflammatory response.

#### Variances of Glycopeptide Ratios Between Patients Having DR With or Without DME

DME is a complication that may occur at any stage of DR. Abnormal fluid accumulation in the macular area leads to increased macular thickness, resulting in DME (20). DME is usually vision-threatening and may impose a financial burden on patients due to the expensive anti-VEGF treatment. However, the causes of macular edema remain unclear. Comparing the differences between DME(+) and DME(−) patients will enable clinicians to better understand the pathogenesis of DME and help identify high-risk populations.

At baseline, 68 patients in NPDR and PDR groups were diagnosed with DME while 45 patients were not. Analysis using the Mann-Whitney U test revealed that DSIgG1: G2F/G1F; DSIgG2: G1FN/G0FN, G2F/G0F, and G2F/G1F were significantly lower in the DME(+) group compared to the DME(−) group as shown in Figure 3. This implies that DR patients with DME exhibit higher levels of inflammatory response.

### Diagnostic Performance of DSIgG N-Glycopeptide Ratio Models

**Fig. 4:**
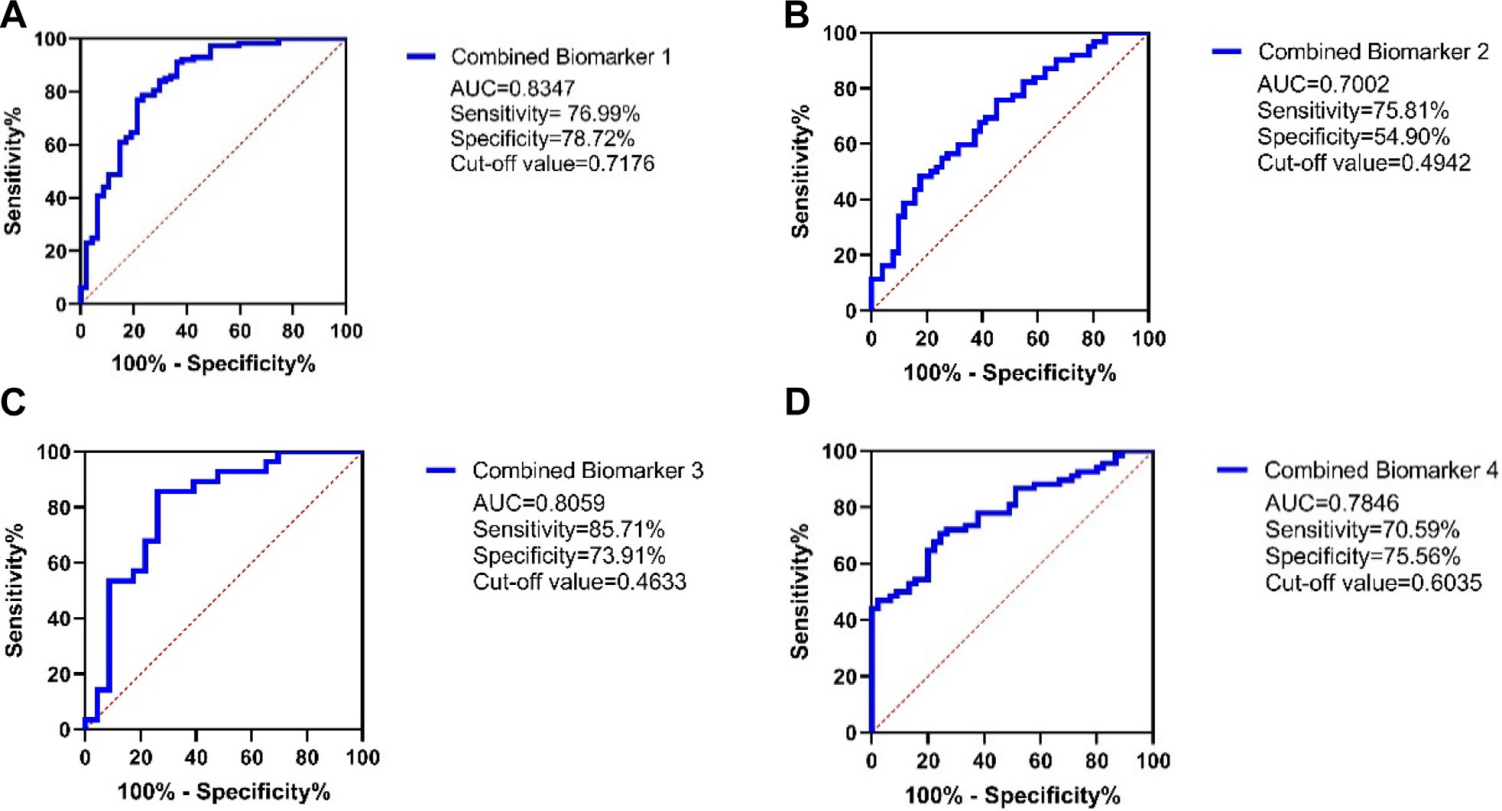
**ROC curves.** AUC–ROC was used to indicate diagnostic efficacy. *A*, combined biomarker 1 aimed to distinguish NDR patients from DR patients. DSIgG1: G1F/G0F, G1FN/G0FN, G2F/G0F; DSIgG2: G1F/G0F, G1FN/G0FN, G2/G1, G2F/G0F, G2F/G1F were included in this anaylsis. *B*, combined biomarker 2 aimed to distinguish NPDR patients from PDR patients. DSIgG1: G1FN/G0FN, G1FN/G1F, G2F/G0F, G2F/G1F; DSIgG2: G0FN/G0F, G1FN/G1N, G2F/G0F, G2F/G1F, G2F/G2 were included in this anaylsis. *C*, combined biomarker 3 aimed to distinguish mild and moderate NPDR patients from severe NPDR patients. DSIgG1: G1FN/G0FN, G1FN/G1F, G2F/G0F, G2F/G1F; IgG2: G1F/G1, G2F/G1F were included in this anaylsis. *D*, combined biomarker 4 aimed to distinguish DR patients with or without DME. DSIgG1: G0FN/G0F, G1F/G0F, G1FN/G1F, G2F/G0F, G2F/G1F; DSIgG2: G1FN/G0FN, G2/G1, G2F/G0F, G2F/G1F were included in this anaylsis. AUC–ROC, area under the receiver operating characteristic curve; DME, diabetic macular edema; DR, diabetic retinopathy; NDR, non-diabetic retinopathy; NPDR, nonproliferative diabetic retinopathy; PDR, proliferative diabetic retinopathy; ROC, receiver operating characteristic.

Four logistic regression models consisting of multiple DSIgG N-glycopeptide ratios with superb discriminative capability were selected to construct a diagnostic model which is shown in Figure 4. Combined Biomarker 1, consisting of eight glycopeptide ratios, achieved a high accuracy (AUC = 0.8347, sensitivity = 76.99%, specificity = 78.72%) when discriminating NDR patients from DR patients. Nine DSIgG N-glycopeptide ratios were included in Combined Biomarker 2, which distinguished NPDR from PDR patients with excellent performance (AUC = 0.7002, sensitivity = 75.81%, specificity = 54.90%). Combined Biomarker 3 used six DSIgG N-glycopeptide ratios to successfully determine whether patients had mild and moderate NDPR or severe NPDR (AUC = 0.8059, sensitivity = 85.71%, specificity = 73.91%). Lastly, Combined Biomarker 4 consists of nine DSIgG N-glycopeptide ratios (AUC = 0.7846, sensitivity = 70.59%, specificity = 75.56%) and performed superbly in identifying the presence of DME in patients.

## Discussion

Diabetic retinopathy is a severe complication that may occur at any stage of DM. Because of the insidious nature of its symptoms, some patients only seek medical support from ophthalmologists at a progressive stage, leading to a grave prognosis and irreversible vision impairment. Thus, it is of great importance to discover easy and accessible measures to screen patients with a high tendency of DR and disease progression.

IgG glycans have exceptional temporal stability, thus having great potential in disease diagnosis. IgG Glycans are important in the development, selection, and maturation of B cells. Changes in IgG glycosylation affect B cell signal transduction through BCRs and CD22 inhibitory receptors (21). IgG autoantibodies can cross-link IgG-specific cellular Fc γ receptors (FcγRs) that are present in most innate immune effector cells, including neutrophils, mast cells, and macrophages (22). FcγRs mediates important IgG effector functions. For example, IgG Fc can induce antibody-dependent cell-mediated cytotoxicity (ADCC) and antibody-dependent cell-mediated phagocytosis (ADCP). Besides that, the Fc region can bind to C1q, leading to the activation of the classical complement pathway and mediating cell lysis (23).

IgG Fc fucosylation is a significant proinflammatory alteration. Lack of core Fuc residue leads to carbohydrate-carbohydrate and carbohydrate-protein interactions between the fucosylated Fc N-glycans and FcγRIIIa glycans, increasing IgG affinity for human FcγRIIIa and FcγRIIIb receptors which in turn enhances ADCC (24). Increased galactosylation in IgG is linked to low inflammatory activity. Galactosylation has been shown to have an impact in binding to FcγRIIa, FcγRIIb, FcγRIIIa, and FcγRIIIb (25). Sialylation has an anti-inflammatory effect by enhancing binding to FcγRIIs and triggering anti-inflammatory signaling (26). The addition of bisecting GlcNA is a proinflammatory alteration (27). Bisecting GlcNAc prevents the addition of a core Fuc residue, which plays an important role in proinflammation (28).

IgG N-glycosylation is closely related to quite a few diseases, including DR. A study found that IgG N-glycosylation was associated with DR progression (18). Another study discovered that IgG glycans were significantly associated with DR (29). In addition, our previous study proved that measures of IgG N-glycosylation were an effective tool in distinguishing patients with NPDR from those with NDR (12). However, these studies are limited due to the absence of a subtype categorization of DR and relatively small sample sizes.

This study brings forward several significant innovations. First, there were hardly any previous studies in the field of DR research that focused on intact glycopeptides analysis, largely due to the low levels of intact glycopeptides and the difficulty of detecting them (30). In this study, we utilized MALDI-ToF-MS to investigate enriched DSIgG N-glycopeptides in NDR, PDR, and NPDR samples and achieved high precision in detecting N-glycopeptides while suppressing impure peaks. Second, compared to previous studies, the sample population in this study was larger and represented patients with diverse clinical characteristics. A total of 160 patients were enrolled and were categorized into three groups by clinical diagnosis: NDR (n = 47), NPDR (n = 51) and PDR (n = 62). Third, the significant differences revealed among subgroups of DR patients add to the current understanding of DR mechanisms and create a strong foundation for research in the future. Age and DM disease duration were found to be correlated with DSIgG N-glycopeptide ratio. Nine DSIgG N-glycopeptide ratios were found to be significantly different among the NDR, NPDR, and PDR groups in this study. Surprisingly, patients with NPDR showed a higher tendency of inflammation compared to patients with PDR. Previous studies found that age and inflammation both lead to a decrease in IgG galactosylation (31), but this occurs most quickly at the onset of inflammatory reactions (32), which could explain why patients with NPDR exhibited higher inflammatory levels than PDR patients. Finally, the logistic regression models we constructed based on DSIgG N-glycopeptides exhibited good discriminatory power for determining whether patients have DR or NDR (AUC = 0.8347), NPDR or PDR (AUC = 0.7002), mild/moderate or severe NPDR (AUC = 0.8059), and the presence of DME (AUC = 0.7846). Thus, we conclude that DSIgG N-glycopeptides are accurate, accessible, and convenient potential biomarkers that may be applied in future clinical practice.

There are a few limitations in our study. First, since this was exploratory research, the sample size was ideal compared to similar research but should be further expanded in subsequent studies. Second, although this was a multicentric study, our research mainly focuses on the Chinese population and lacks representation of ethnic groups outside of this population.

## Conclusion

In this multicenter research, mass spectrometry was used to explore the relationship between DSIgG N-glycosylation and the course of DR. Significant differences were found between NDR, NPDR, and PDR groups, and also between patients with mild, moderate, and severe NPDR. The difference in the ratios between patients with or without DME was statistically significant as well. Moreover, the four logistic regression models we constructed based on DSIgG N-glycopeptides exhibited good discriminatory power. Together, the logistic regression models form a diagnostic model that accurately informs whether a patient has DR or NDR, NPDR or PDR, mild/moderate or severe NPDR, and whether DME is present. It may be applicable in future clinical practice and can deepen clinicians’ understanding of the pathophysiology of diabetic retinopathy.

## Data Availability

The mass spectrometry proteomics data are available via ProteomeXchange with identifier PXD057612.

## Supporting information

This article contains supporting information.

## Conflict of interest

The authors declare that they have no conflicts of interest with the contents of this article.
