# Supplementary material for: Serum Disease-Specific IgG Fc Glycosylation as Potential Biomarkers for Nonproliferative and Proliferative Diabetic Retinopathy Using Mass Spectrometry
Source: Mol Cell Proteomics. 2025 Apr 9;24(5):100967. doi: 10.1016/j.mcpro.2025.100967 (PMC12148564; doi:10.1016/j.mcpro.2025.100967)
Supplement: Supplementary Materials (figure) [file mmc1.docx]

**Supplementary** **materials for:**

**Serum Disease-specific IgG Fc Glycosylation as Potential Biomarkers for Nonproliferative and Proliferative Diabetic Retinopathy Using Mass Spectrometry**

Yishuang Mao^a,1^; Jiyun Zhang^b,1^ Yixin Zhang^c,1^; Bojie Hu^d^; Yuhua Hao^e^; Zhonghao Yuan^b^; Xufeng Zhao^a^; Yusong Wang^a^; Zhangwanyu Wei^a^; Weihong Yu^a,f,*^; Zhili Li^b,* *^;

^a^ Department of Ophthalmology, Peking Union Medical College Hospital, Beijing, China.

^b^ Department of Biophysics and Structural Biology, Institute of Basic Medical Sciences, Chinese Academy of Medical Sciences & School of Basic Medicine, Peking Union Medical College, China.

^c^ Beijing Tongren Eye Center, Beijing Tongren Hospital of Capital Medical University

^d^ Department of Retina, Tianjin Medical University Eye Hospital, Tianjin, China.

^e^ Department of Ophthalmology, The Fourth Hospital of Hebei Medical University, Shijiazhuang, China

^f^ Key Laboratory of Ocular Fundus Diseases, Chinese Academy of Medical Sciences, Beijing, China.

**^*^Corresponding author: Weihong Yu**, Department of Ophthalmology, Peking Union Medical College Hospital, No.1 Shuaifuyuan Road, Dongcheng District, Beijing 100730, China. Tel:+86-10-69156351; E-mail: yuweihongpumch@163.com

**^**^Corresponding author:** **Zhili Li**, Department of Biophysics and Structural Biology, Institute of Basic Medical Sciences, Chinese Academy of Medical Sciences & School of Basic Medicine, Peking Union Medical College, No.5 Dongdan San Tiao, Beijing 100005, China. Tel/Fax: +86-10-69156479; E-mail: lizhili@ibms.pumc.edu.cn

^1^These authors contributed equally to this work.

**Funding**

This study was supported by Beijing natural science foundation-Beijing Tianjin Hebei basic research cooperation project [No. J200006]; Beijing Natural Science Foundation [No. 7244380].

Supplementary Figure

Figure S1. Representative mass spectra of the DSIgG Fc glycopeptides.

| 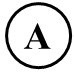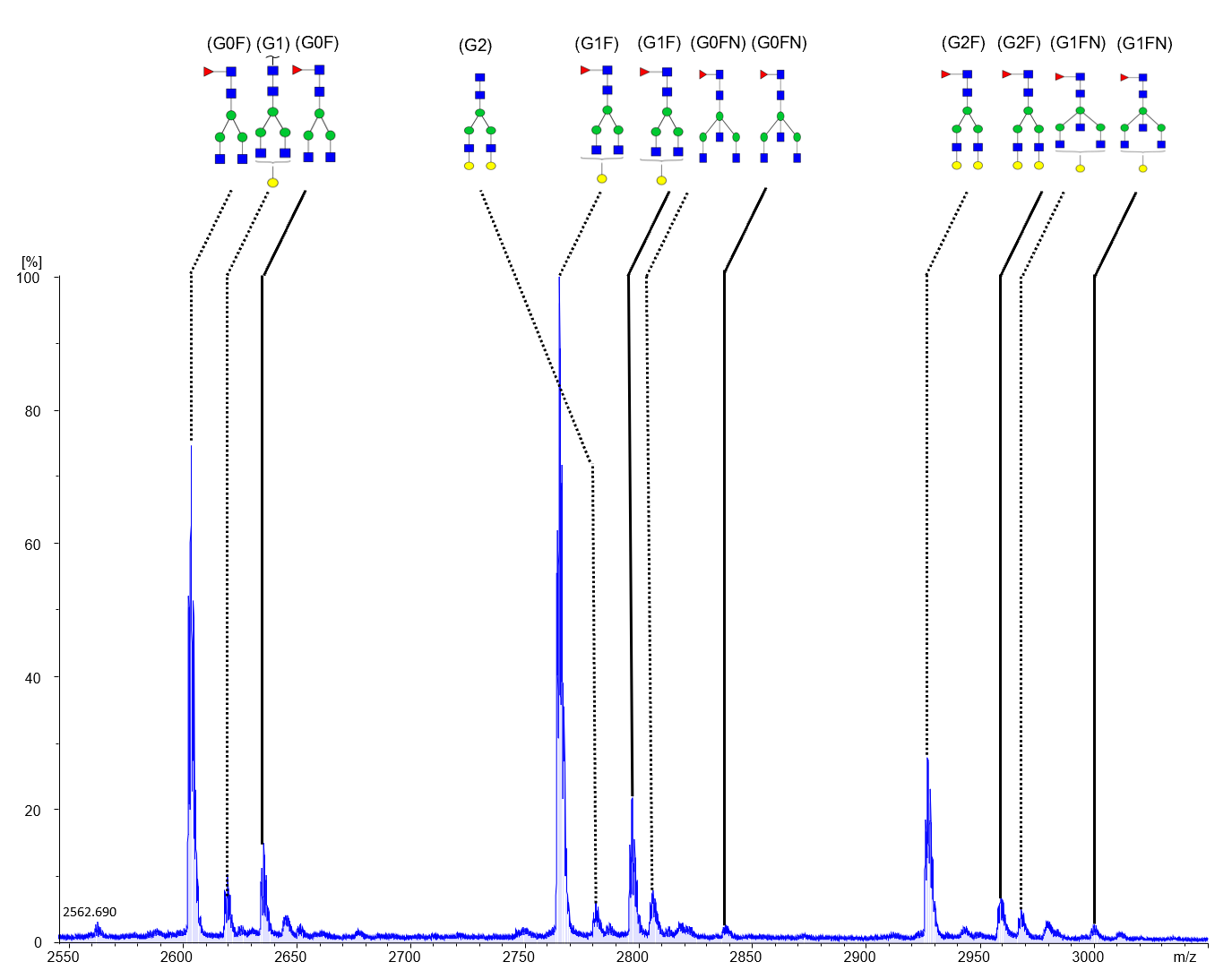 | 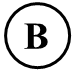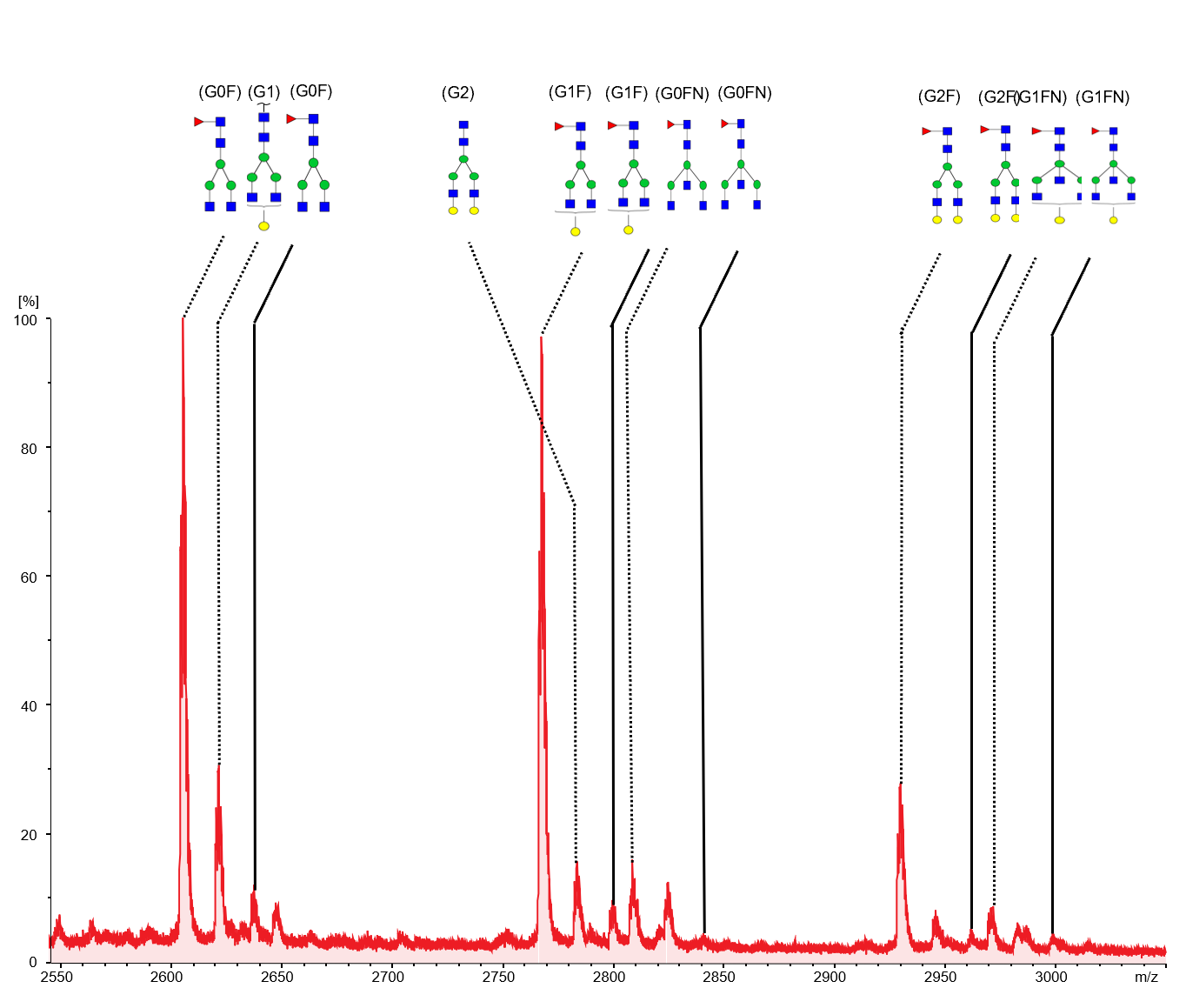 | 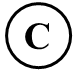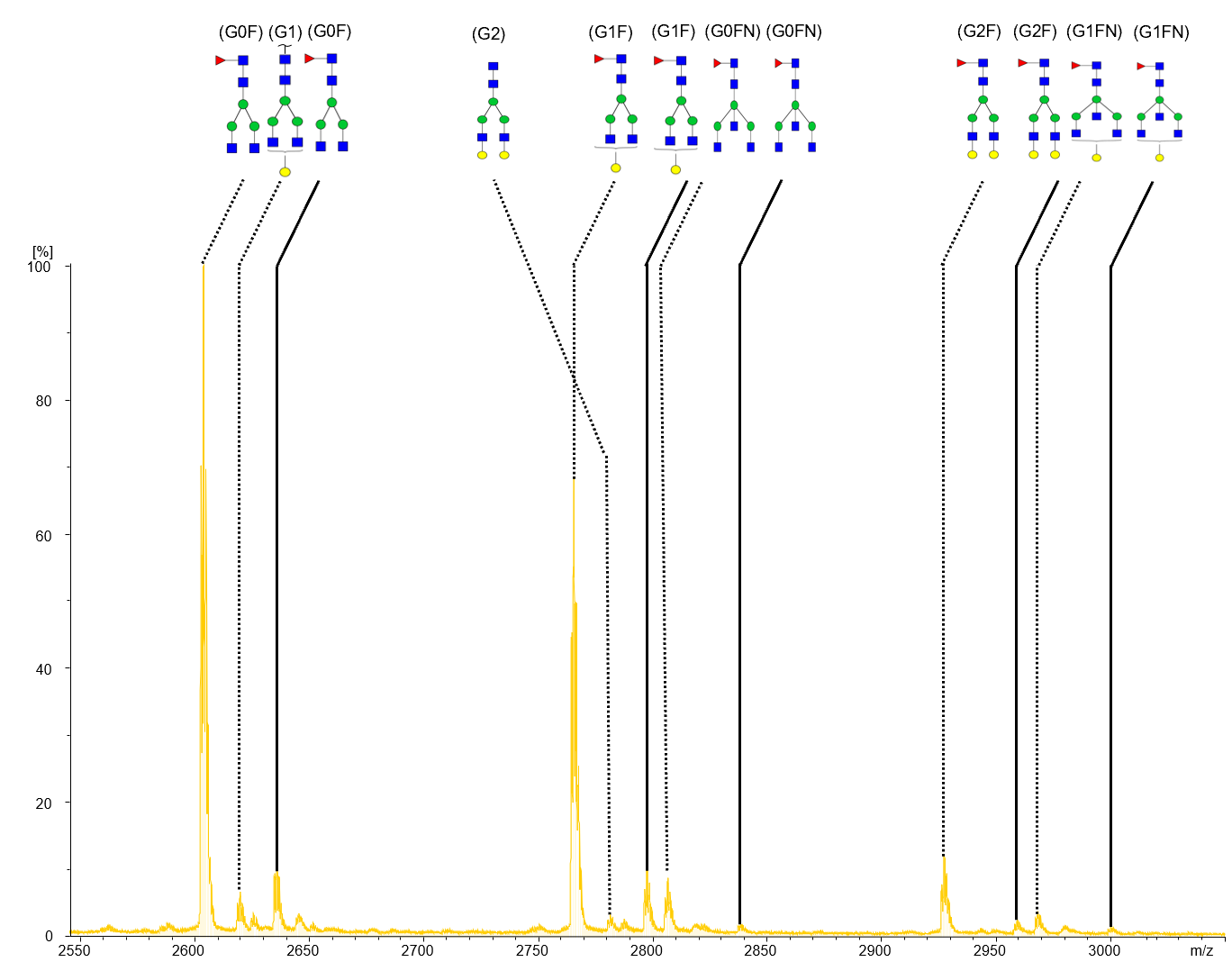 |
| --- | --- | --- |
| DSIgG1 glycopeptides DSIgG2 glycopeptides | | 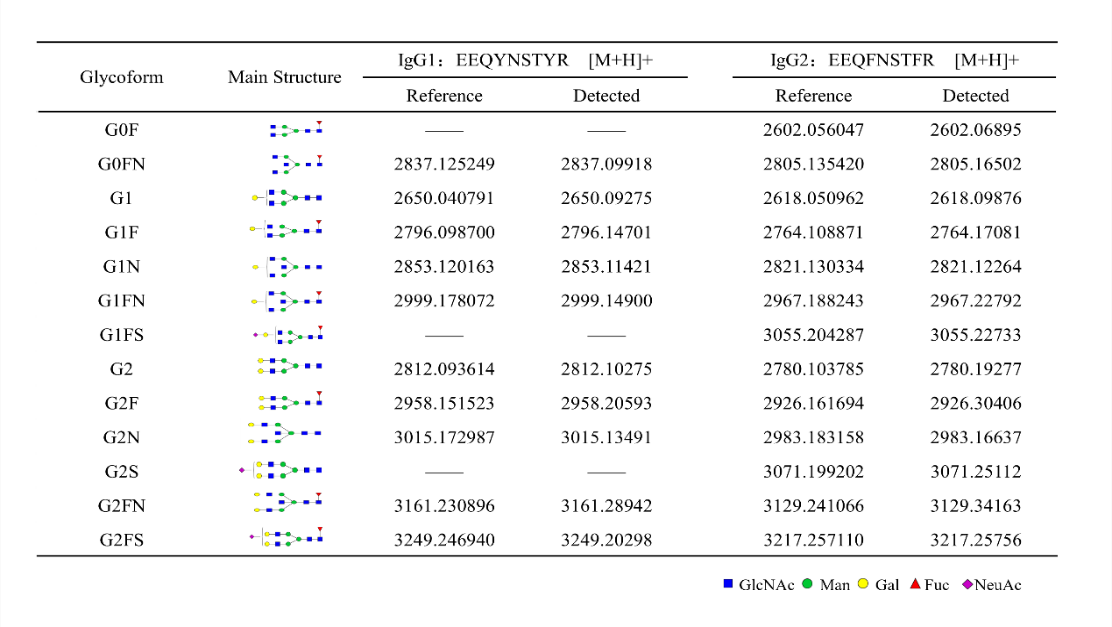 |
| Figure S1. Representative mass spectra of the DSIgG Fc glycopeptides  A) A patient with NDR; B) A patient with NPDR; C) A patient with PDR. | | |

Figure S2. Correlation analysis between glycopeptide ratios in NDR, NPDR and PDR patients.

| 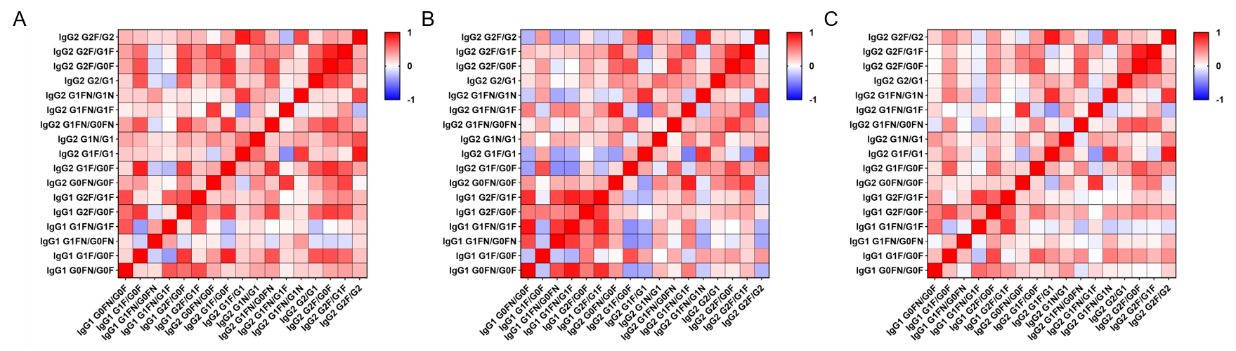 |
| --- |
| Figure S2. Correlation analysis between glycopeptide ratios in NDR (A), NPDR (B) and PDR (C) patients. |
